# Monocrotophos Induced Apoptosis in PC12 Cells: Role of Xenobiotic Metabolizing Cytochrome P450s

**DOI:** 10.1371/journal.pone.0017757

**Published:** 2011-03-21

**Authors:** Mahendra Pratap Kashyap, Abhishek Kumar Singh, Vivek Kumar, Vinay Kumar Tripathi, Ritesh Kumar Srivastava, Megha Agrawal, Vinay Kumar Khanna, Sanjay Yadav, Swatantra Kumar Jain, Aditya Bhushan Pant

**Affiliations:** 1 Indian Institute of Toxicology Research, Lucknow, India; 2 Council of Scientific and Industrial Research, New Delhi, India; 3 Department of Biotechnology, Jamia Hamdard University, New Delhi, India; Johns Hopkins School of Medicine, United States of America

## Abstract

Monocrotophos (MCP) is a widely used organophosphate (OP) pesticide. We studied apoptotic changes and their correlation with expression of selected cytochrome P450s (CYPs) in PC12 cells exposed to MCP. A significant induction in reactive oxygen species (ROS) and decrease in glutathione (GSH) levels were observed in cells exposed to MCP. Following the exposure of PC12 cells to MCP (10^−5^ M), the levels of protein and mRNA expressions of caspase-3/9, Bax, Bcl_2_, P^53^, P^21^, GSTP1-1 were significantly upregulated, whereas the levels of Bclw, Mcl1 were downregulated. A significant induction in the expression of CYP1A1/1A2, 2B1/2B2, 2E1 was also observed in PC12 cells exposed to MCP (10^−5^ M), whereas induction of CYPs was insignificant in cells exposed to 10^−6^ M concentration of MCP. We believe that this is the first report showing altered expressions of selected CYPs in MCP-induced apoptosis in PC12 cells. These apoptotic changes were mitochondria mediated and regulated by caspase cascade. Our data confirm the involvement of specific CYPs in MCP-induced apoptosis in PC12 cells and also identifies possible cellular and molecular mechanisms of organophosphate pesticide-induced apoptosis in neuronal cells.

## Introduction

Organophosphorus (OP) group of pesticides have been used extensively across the world for more than fifty years [1] resulting annual exposure to 2–3 million people [2]. OPs are known to induce acute and chronic neurotoxicity in mammalians primarily by inhibiting acetylcholinesterase (AChE) activity [3], [4]. However, neurotoxicity of OPs has also been reported to link with necrosis [5], apoptosis [6], [7], and oxidative stress mediated pathways [7], [8]. OPs have also been found to induce oxidative stress in developing brain, leading to alter the expression and functions of antioxidant genes [9]. Most of the OPs do not produce the same pattern of behavioral deficits or toxic responses, in part, because of the involvement of different toxicological mechanisms that contribute to the net adverse outcomes [10]. The toxic responses of OPs on cellular and molecular level have been explored in cultured cells using standard endpoints of cytotoxicity and genotoxicity [5], [11]. However, the knowledge on specific pathway(s) involved for individual OP-induced toxicity is needed to be elaborating completely. The involvement of different CYPs has been suggested in the process of oxidative stress [12], mutagenicity [13], apoptosis [14], [15], and behavioural deficits [16]. Significant induction in the expression of different CYPs has been reported in liver exposed to structurally unrelated chemicals [16]. Although, liver is known to be a primary site for CYPs-mediated metabolism, but the expression and inducibility of CYPs in extrahepatic systems such as blood and brain have also been reported [16], [17]. Involvements of the several CYPs in the metabolic activation of drugs and chemicals have also been reported in primary cultures of rat brain neuronal and glial cells [18]. CYPs facilitate biotransformation of xenobiotics by oxidizing them result the formation of number of reactive oxygenated intermediates (ROMs). ROMs are highly unstable in nature, but their presence for short duration in the cells may lead cellular damages [19], [20]. ROMs-induced damages have been suggested to cause abrupt xenobiotic metabolism as well as the formation of more hazards intermediates, which could ultimately lead hyper-mutability, genomic instability, adverse effects on number of proteins related to cell cycle checkpoints and neuronal cell death [21].

Thus, we studied apoptotic changes and their correlation with expression of selected cytochrome P450s (CYPs) in PC12 cells exposed to MCP. MCP was selected as model pesticide, since it has been used extensively worldwide and is known for its neurotoxicity [22], [23]. PC12 cells were selected because of known expressions of CYPs [24] and most of the marker associated with neuronal structures, functions, toxicity and repair [9], [25]

## Results

### Intracellular glutathione levels

**Figure 1:**
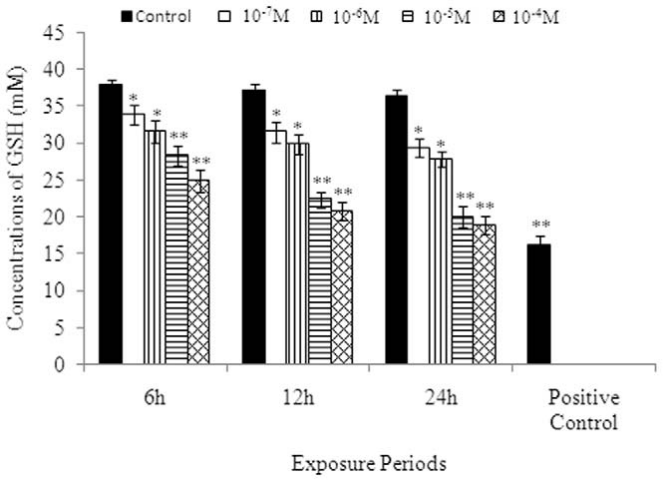
Glutathione (GSH) levels in PC12 cells exposed to MCP (10^−4^–10^−7^ M) for 6, 12, and 24 h assessed by using fluorescence based Glutathione Detection Kit (Catalog no. APT250, Chemicon, USA). To estimate the GSH levels, the lysed samples (90 µl/well) were transferred to 96 well black bottom plates and mixed with freshly prepared assay cocktail (10 µl) containing monochlorobimane (MCB), a dye has high affinity for glutathione in cells compared to other thiols. Plates were read at excitation wavelength 380 nm and emission wavelength 460 nm after the incubation for 1–2 h at 37°C by using Multiwell Microplate Reader (Synergy HT, Bio-Tek, USA). Standard curve was plotted using the glutathione standard supplied in the kit and used to calculate the experimental values. The data are expressed in intracellular concentrations of GSH±SEM, n = 3. *  = P<0.05, ** = p<0.001.

Data of MCP-induced alterations in the levels of intracellular GSH concentrations are summarized in figure 1. Statistically significant (p<0.001) decrease in the values were observed at 6, 12, and 24 h exposures, i.e., 31.4±1.5 mM, 29.7±1.3 mM, and 27.8±1.1 mM following an exposure of MCP (10^−6^ M) and 28.2±1.3 mM, 22.3±1.1 mM, and 19.9±1.4 mM in cells exposed to MCP (10^−5^ M) when compared with unexposed controls i.e., 37.8±0.8 mM (6 h), 37.1±1.0 mM (12 h) and 36.3±0.9 mM (24 h) respectively.

### ROS generation

**Figure 2:**
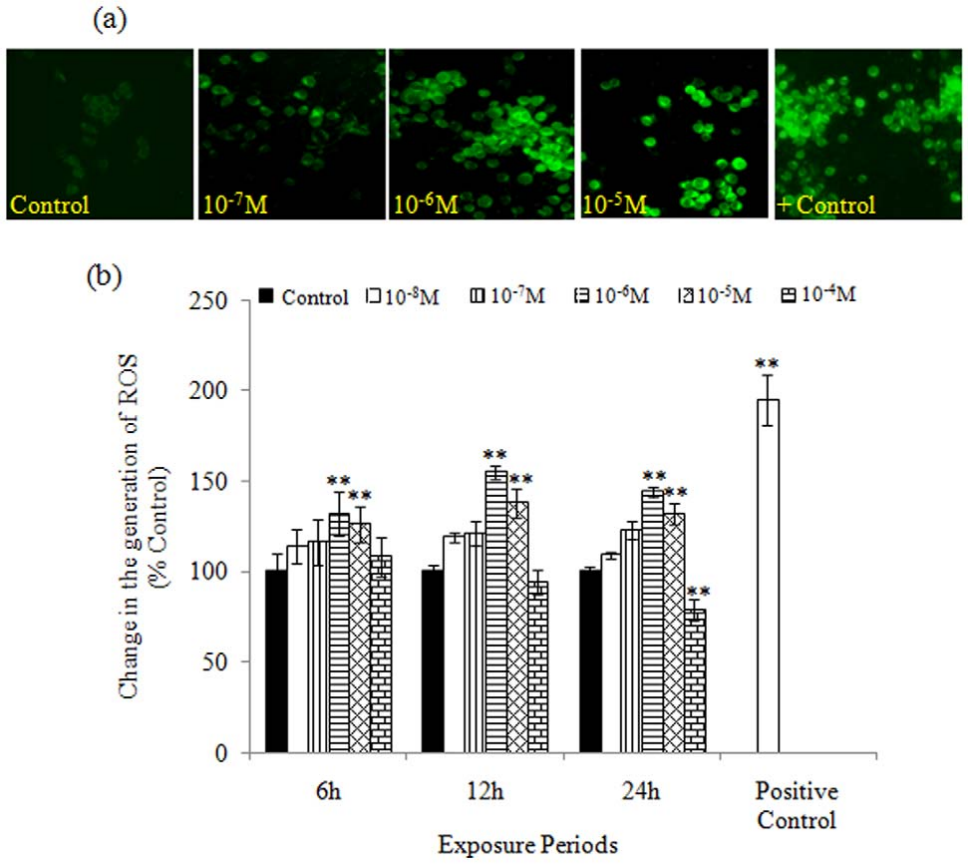
Reactive Oxygen Species (ROS) generation in PC12 cells exposed to MCP. (a) Representative microphotographs showing MCP-induced reactive oxygen species (ROS) generation in PC12 cells. ROS generation was studied using dichlorofluorescin diacetate (DCFH-DA) dye. Images were captured by Nikon phase contrast cum fluorescence microscope (model 80i) attached with 12.7 Megapixel Nikon DS-Ri1 digital CCD cool camera. (b) Percent change in ROS generation following 6, 12 and 24 h exposure of various concentrations of MCP in PC12 cells assessed by spectrofluorometric analysis. In brief, cells (1×10^4^ per well) were seeded in poly L-lysine pre-coated 96 well black bottom culture plates and allowed to adhere for 24 h in 5% CO_2_–95% atmosphere at 37°C. Cells were exposed to MCP (10^−4^ to 10^−8^ M) for 6, 12 and 24 h. Following the exposure, cells were re-incubated with 2′, 7′ dichlorodihydrofluorescein-diacetate (DCFH-DA) (20 µM) for 30 min at 37°C and fluorescence intensity was measured using multiwall micro plate reader (Synergy HT, Bio-Tek, USA) on excitation wavelength at 485 nm and emission wavelength at 528 nm. The data are expressed in mean of percent of the unexposed control ± SEM, n = 8. *  = P<0.05, ** = p<0. 001.

MCP (10^−6^ M and 10^−5^ M) induces significant ROS production in PC12 cells at all the incubation periods, i.e., 132±11% and 116±10% (6 h); 155±3.6% and 138±7.9% (12 h), and 144±2.7% and 169±5.6% (24 h) respectively. ROS production was insignificant following 10^−7^ M and 10^−8^ M concentrations of MCP at all the time points. While, MCP (10^−4^ M) exposure for 12 and 24 h was significantly cytotoxic (Figure 2b). Fluorescence microscopic analysis using DCFH-DA fluorescence dye maintains the linearity with the data obtained by spectro-fluorimetric analysis (Figure 2a).

### Apoptosis detection

**Figure 3:**
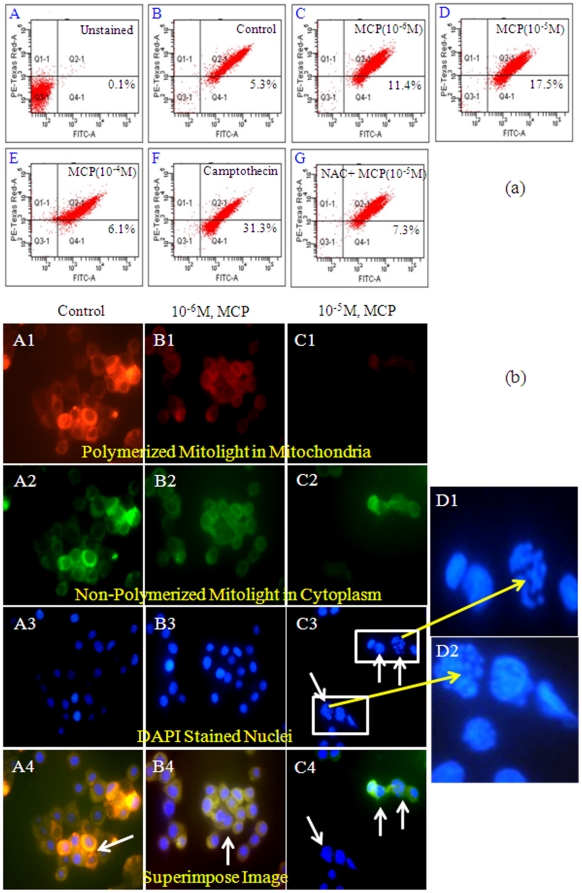
Apoptosis induction in PC12 cells exposed to MCP. (a) Apoptosis detection in PC12 cells exposed to MCP using Mitolight™ apoptosis detection kit (catalog no. APT142, Chemicon, USA). (A) Unstained cells; (B) Control cells; (C) PC12 cells exposed to MCP (10^−6^ M) for 6 h; (D) PC12 cells exposed to MCP (10^−5^ M) for 6 h; (E) PC12 cells exposed to MCP (10^−4^ M) for 6 h; (F) Experimental positive control- PC12 cells exposed to campothecin (3 µg/ml) for 6 h; (G) Cells pretreated with 10 µM NAC for 1 h and then exposed with MCP(10^−5^ M) for 6 h. (b) Apoptosis detection by Mitolight™ apoptosis detection kit using Upright Phasecontrast Microscope (Nikon 80i, Japan) at 10×100x oil immersion magnification. The images were snapped by Nikon DS-Ri1 (12.7 megapixel) camera. Figure A1- Control cells showing intense red color due to polymerization of Mitolight dye in mitochondria indicative of healthy mitochondria. Figure A2- green color indicates the accumulation of non-polymerized dye in cytoplasm. Figure A3- Nuclei stained with DAPI.Figure A4- Superimposed microphotographs showing healthy mitochondria with intact membrane. Figure B1-B4: PC12 cells exposed to MCP (10^−6^ M) for 6 h shows significant dissipation in Mitochondrial membrane potential. Figure C1–C4: PC12 cells exposed to MCP (10^−5^ M) for 6 h. C-3: cells showing nuclear condensation and fragmentations (D1 and D2 are magnified view highlighting the same). C-4: Superimposed microphotograph showing apoptotic events.

MCP (10^−5^ M) exposure for 6 h induces significant (3 fold of control) apoptosis in PC12 cells. While the magnitude of apoptosis induction was low (2 fold of control) in cells exposed to MCP (10^−6^ M) for 6 h. Increase in necrosis and decrease in apoptosis was observed in cells exposed to MCP (10^−4^ M) for 6 h. System optimization was confirmed by induction of apoptosis in 31.3% cell population following camptothecin (3 µg/ml) exposure for 6 h, and served as positive control (Figure 3a). Fluorescence imaging of the cells kept under identical experimental conditions confirms our data obtained by FACS analysis (Figure 3b).

### Bis-benzimide Staining

**Figure 4:**
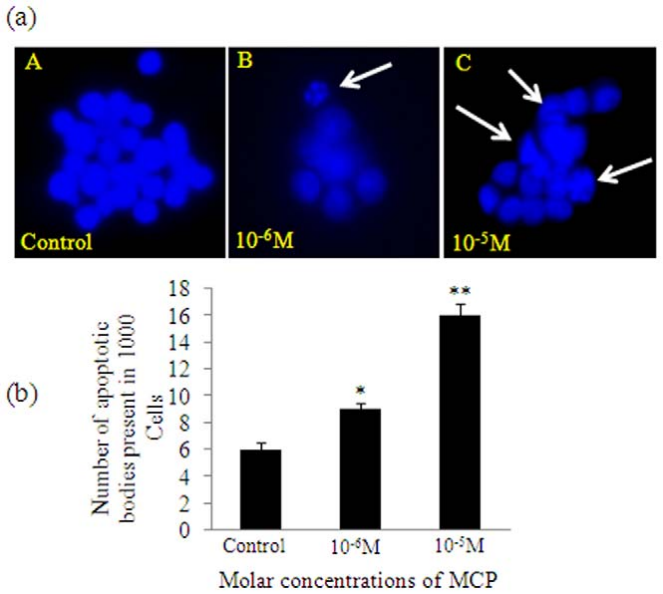
DAPI staining for the detection of MCP-induced apoptosis. (a) Representative microphotographs showing induction of Apoptosis in PC12 cells exposed to various concentrations of MCP for variable time periods. (A): Unexposed control cells (B): cells exposed to 10^−6^ M MCP showing apoptotic body; (C): Cells exposed to 10^−5^ M MCP Showing more damages. (b) Apoptosis induction in PC12 cells exposed to various concentrations of MCP for different time periods. Apoptotic Bodies were counted by using Upright phase contrast Fluorescent microscope (Nikon 80i, Japan) at 10×100x oil immersion magnification and images were grabbed by Nikon DS-Ri1 (12.7 megapixel) camera. Minimum 1000 cells were counted in each slide in triplicate. * p<0.05, **p<0.001

Nuclear condensation and DNA fragmentation were studied as markers of apoptosis in PC12 cells following the exposure of selected concentrations of MCP. Findings of the assays were showing the similar trends as observed in case of MMP and responded to MCP insult in a dose dependent manner in PC12 cells (Figure 4a & b).

### Transcriptional changes

**Figure 5:**
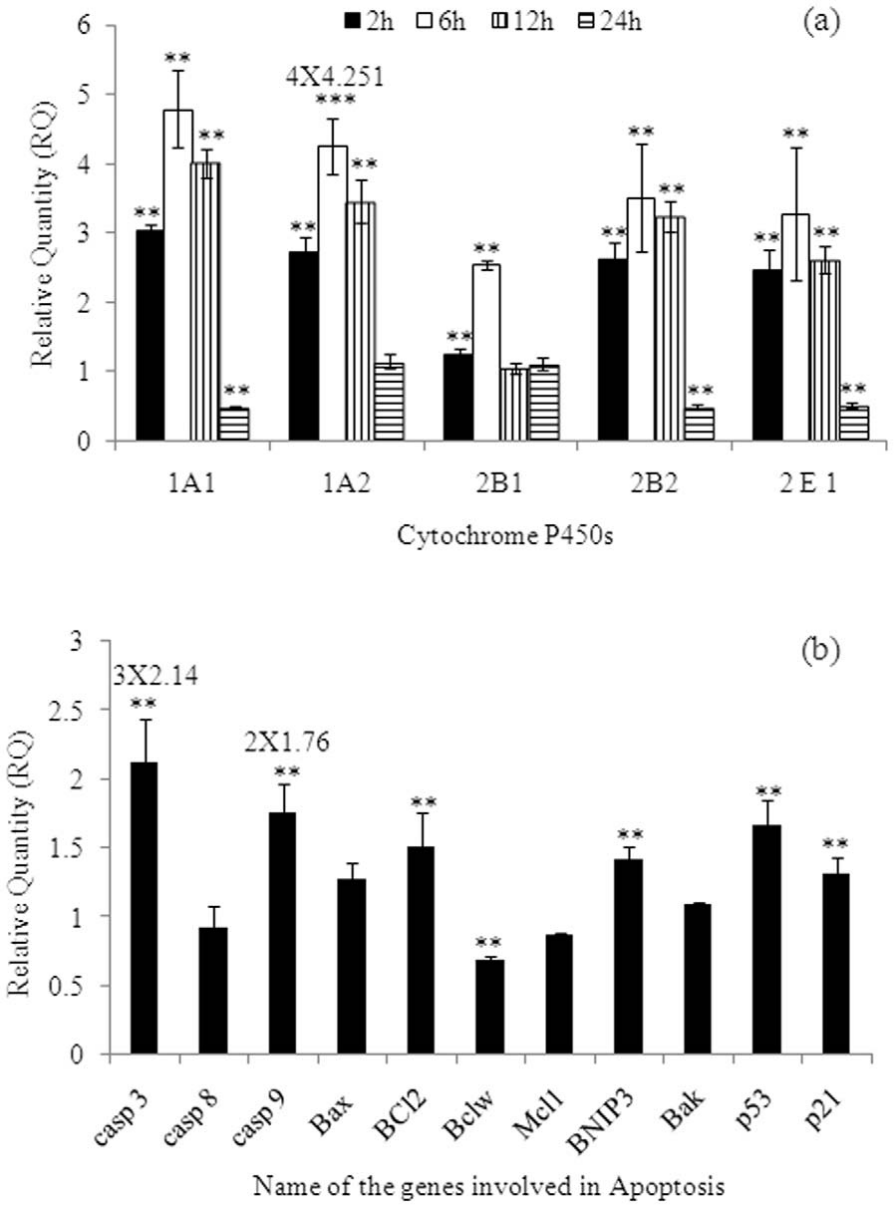
Transcriptional changes in the levels of selected xenobiotic metabolizing cytochrome P450s (CYPs) and apoptosis markers in PC12 cells exposed to MCP. (a) MCP-induced alterations in the mRNA expression of marker genes associated with metabolism of xenobiotics in PC12 cells. Quantitative Real Time PCR (RT-PCR^q^) was performed in triplicate by TaqMan Probe using ABI PRISM® 7900HT Sequence Detection System (Applied Biosystems, USA). Actin-β was used as internal control to normalize the data and MCP induced alterations in mRNA expression are expressed in relative quantity compared with respective unexposed control groups. (b) MCP induced alterations in the mRNA expression of marker genes associated with apoptosis in PC12 cells. Quantitative Real Time PCR (RT-PCR^q^) was performed in triplicate by SYBR Green dye using ABI PRISM® 7900HT Sequence Detection System (Applied Biosystems, USA). Actin-β was used as internal control to normalize the data and MCP induced alterations in mRNA expression are expressed in relative quantity (RQ) compared with respective unexposed control groups. Reliability of Specific products was checked by melting curve analysis as well as running the product onto 2% agarose Gel.

MCP (10^−5^ M) exposure for 2, 6, 12, and 24 h induces significant alterations in the expression levels of mRNA of CYP 1A1, 1A2, 2B1, 2B2, and 2E1. In a biphasic response, at 2 and 6 h, expression was increased and thereafter levels were decreased at 12 and 24 h. However, at 12 h, values were significantly higher than unexposed control cells for all the CYPs except for CYP2B1. Interestingly, the peak levels of all the CYPs were observed in cells exposed for 6 h i.e., 4.79±0.56; 17.00±1.63; 2.54±0.07; 3.52±0.77; and 3.28±0.95 fold of control for CYP 1A1, 1A2, 2B1, 2B2, and 2E1 respectively. The elevated levels of mRNA were almost restored to basal level rather below to that by 24 h exposure (Figure 5a).

Since, MCP (10^−5^ M) exposure for 6 h was found to be most effective in the induction of mRNA expression of CYPs, the mRNA expression study for the genes associated with apoptosis was restricted for 6 h only. Results show significant up-regulation in the expression of mRNA for Caspase-3 (6.37±0.31), Caspase-9 (3.51±0.21), Bax (1.28±0.12), Bcl2 (1.50±0.25), Bnip3 (1.43±0.08), p53 (1.67±0.17), and p21 (1.31±0.12) fold of control, whereas down regulation was observed in case of Bclw (0.69±0.03), and Mcl1 (0.88±0.01) fold of control (Figure 5b).

### Western blot analysis

**Figure 6:**
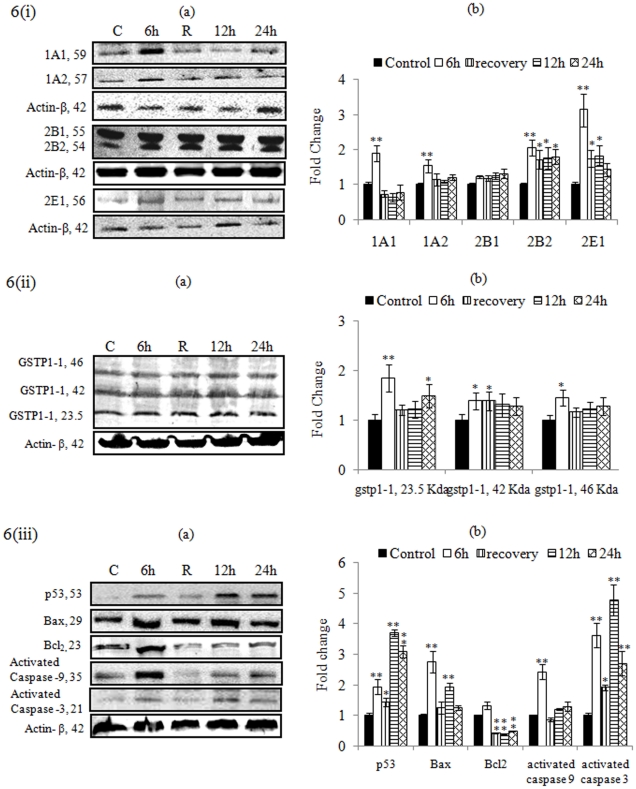
Alterations in the expression of proteins involved in the metabolism [figure-6 (i) a & b], oxidative stress [figure-6 (ii) a & b], and cell death [figure-6 (iii) a & b] were studied in PC12 cells exposed to MCP (10^−5^ M) for various time periods. Actin- β was used as loading control to normalize the data. (a) Lane (A): untreated control; (B): Cells exposed to MCP for 6 h; (C): Proteins isolated after 24 h, i.e., 6 h of MCP exposure +18 h without exposure (auto-recovery period); (D): Cells exposed to MCP for 12 h; (E): Cells exposed to MCP for 24 h. (b) Relative quantification of alterations in the expression of different proteins., viz CYP1A1 (59 kDa), CYP1A2 (57 kDa), CYP2B1 (55 kDa), CYP2B2 (54 kDa), CYP2E1 (56 kDa), GSTP1-1 (23.5, 42 and 46 kDa), P^53^ (53 kDa), Bax (29 kDa), Bcl_2_ (23 kDa), activated caspase-9 (35 kDa), activated caspase-3 (21 kDa), and Actin-β (42 kDa) in PC12 cells exposed to MCP (10^−5^ M) for various time periods. Actin-β was used as internal control to normalize the data. Quantification was done in Gel Documentation System (Alpha Innotech, USA) with the help of AlphaEase™ FC StandAlone V.4.0 software. *  = P<0.05, ** = p<0. 001.

MCP (10^−5^ M) exposure for 6 h shows peak upregulation of protein expression of CYP 1A1 (1.89±0.23), 1A2 (1.53±0.19), 2B1 (1.23±0.05), 2B2 (2.06±0.23), 2E1 (3.13±0.47), p53 (1. 94±0.24), GSTP1-1: 23 kda (1.85±0.27), GSTP1-1: 42 Kda (1.39±0.17), GSTP1-1: 46 kda (1.46±0.16), Bax (2.75±0.34), Bcl_2_ (1.33±0.12), activated Caspase-9 (2.43±0.14) and activated Caspase-3 (3.62±0.41) fold of control. Protein expression of CYP1A1/1A2 and Bax came to the basal level in cells exposed to MCP (10^−5^ M) for 12, and 24 h. However, the levels of protein expression of CYP2B2, 2E1, p53 and all forms of GSTP1-1 were higher than unexposed control cells following MCP exposure for 12 and 24 h. In case of Bcl_2_ values were observed below the basal level at 12, and 24 h exposure. Significant restoration of altered levels were observed in recovery group i.e., CYP1A1 (0.74±0.09), CYP1A2 (1.16±0.17), CYP2B1 (1.19±0.08), CYP2B2 (1.72±0.26), CYP2E1 (1.75±0.24), P^53^ (1.27±0.19), Bax (1.18±0.14), Bcl_2_ (0.42±0.02), and activated Caspase-3 (0.86±0.07) [Figure 6 (i) a & b; (ii) a & b; (iii) a & b].

### Immunocytochemical analysis

**Figure 7:**
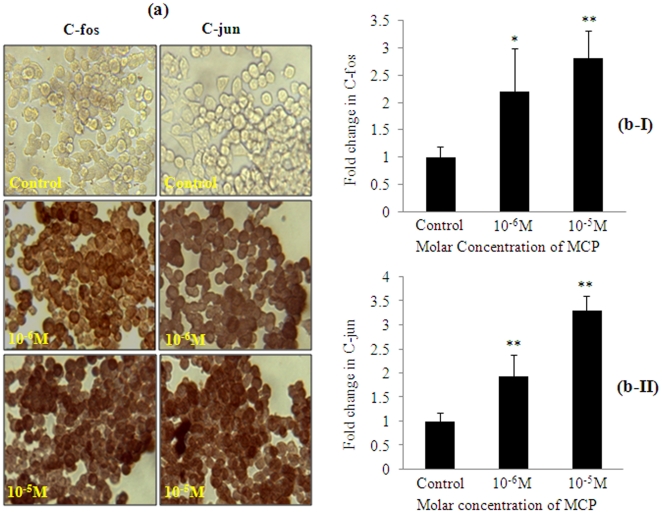
MCP induced alterations in the expression of early response genes. (7a) Representative microphotographs of immunocytochemical localization of C-fos and C-jun proteins in PC12 cells exposed to MCP (10^−5^ and 10^−6^ M). Images were taken by Nikon Eclipse 80i equipped with Nikon DS-Ri1 12.7 megapixel camera, Japan. (7b I & II) Relative quantification of fold inductions in the expression of C-fos and C-jun proteins in PC12 cells exposed to MCP (10^−5^ and 10^−6^ M) for 6 h. Leica Q-Win 500 image analysis software was used to quantify the expression of C-fos and C-jun. Data were calculated as mean ± SE of at least 20 fields from three independent experiments.

MCP (10^−6^ M and 10^−5^ M) exposure for 6 h induces significant (p<0.001) protein expression of C-fos (2.20±0.51 fold, 2.81±0.78), and C-jun (1.93±0.51 fold, 3.30±0.72) fold of control respectively. MCP exposure of 10^−5^ M induces the alteration in the expression with greater magnitude than MCP 10^−6^ M concentration and this magnitude difference was statistically significant (p<0.001) (Figure 7a & b-I, II).

## Discussion

The high lipid contents, high oxygen consumption, and low levels of glutathione contents are suggested reasons for ROS-mediated vulnerability of brain cells against xenobiotics [26]. In the present study, we also observed the significant dose and time dependent induction in ROS generation and decrease in glutathione (GSH) levels, which were found to be associated with apoptotic changes. Earlier we reported the increase of LPO in PC12 cells exposed to MCP [7]. Similar kind of associations have also been reported using cultured cells of neural origin and rat brain slices [10], blood mononuclear cells [27], and mouse macrophage cell lines [28], [29].

The activation of cytochrome P450s and their interaction with mitochondrial chain complexes have been suggested in chemical-induced apoptosis [20], [30]. The involvement of CYPs in organophosphates-induced apoptosis in neuronal cells has also been indicated [31]. However, we are reporting first time that MCP-induced apoptosis and oxidative stress are associated/regulated by specific isoforms of CYPs in PC12 cells. We observed significant induction in the expression of CYPs even at 2 h exposure, which was found to be upstreamed to ROS generation by 6 h in PC12 exposed to MCP. Such induced expression of CYPs in early hours might have played important role in the production of reactive oxygenated molecules (ROMs), which are known to induce ROS generation [32], LPO [33], GSTs [34], and eventually to apoptosis [15], [20]. In the present investigations, apoptosis induction and oxidative stress was found to be associated with upregulation of CYP1A1. Such increased expression of CYP1A1 has also been reported increase the excretion rate of 8-oxoguanine (oxo8Gua) in human hepatoma cell line, a biomarker of oxidative DNA damage [35]. CYP1A1 and CYP1B1 have been demonstrated to catalyze catechol estrogen formations, which play a key role in 2, 3, 7, 8-tetrachlorodibenzo-p-dioxin-induced oxidative damage in cultured human mammary epithelium cells [36]. Induced CYP2E1 was found to cause oxidative stress by depleting the intracellular GSH levels [37], activation of the p38 MAP kinase pathway, and induction of the transcription factor Nrf2 [38], in human hepatoma cell line-HepG2. The role of CYP2E1has been suggested in alcohol-induced oxidative DNA damage in liver of null mice [39].

Induction in the expression levels of CYPs (CYP1A1/1A2, 2B1/B2 and 2E1) were higher at 6 h, which brought down towards the basal level by 24 h. Similarly, apoptotic events were also found to reduce with the passage of time. This could be due to increased necrosis at 12 and 24 h exposures, as discussed in our earlier report too [7]. Since, induced expression of CYPs is regarded as defence mechanism to detoxify the effect of xenobiotics [40], thus, initial increase in the expression (mRNA and protein) of CYPs suggest responsiveness of cells against MCP exposure. Whereas, the decreased levels of CYPs in cells exposed to MCP for longer period might be due to significant necrotic cell death. It has already been demonstrated in case of various xenobiotics that higher doses for low time periods and lower doses for higher time periods can convert apoptosis into necrosis [41].

Following MCP exposure, we observed a significant up-regulation in the expression of immediate early response gene proteins, i.e., C-fos and C-jun. Such significant up-regulation might be due to oxidative stress induced by the massive production of ROS/ROMs or induction of JNK pathway during CYPs-mediated metabolism of MCP. The association of chemical-induced over expression of the various CYPs, and oxidative damage is well established [42]. The induced level of GSH is an indicator of strong anti-oxidant status in cell system [28], whereas, reduced GSH levels were found to be associated with impaired anti-oxidant activities [43]. The lower levels of GSH in brain cells have been reported to facilitate the dissociation of GSTP1-1/JNK complex, and activation of JNK pathway [44]. In the present study, increased expression of GSTP1-1 and decreased GSH levels may also be correlated with the activation of JNK pathway, and subsequent cell death. However, upon the longer exposure, the GSTP1-1 levels came down very near to basal, which indicate either the failure of self defense due to activation of JNK pathway or necrotic cell death. Such GSTP1-1 dependent activation of JNK pathway is well documented in Jurkat [45], human neuroblastoma cell line [46], and in NB4 cell line [44], against variety of chemical exposures.

The other possible reason for our findings might be due to the non-enzymatic direct binding of GSH with CYPs mediated reactive metabolites of MCP. This phenomenon has already been reported in case CYPs mediated metabolism of paracetamol, where the levels of GSH were found to be depleted upon the accumulation of reactive metabolite - N-acetyl-p-benzoquinone imine (NAPQI) [47].

**Figure 8:**
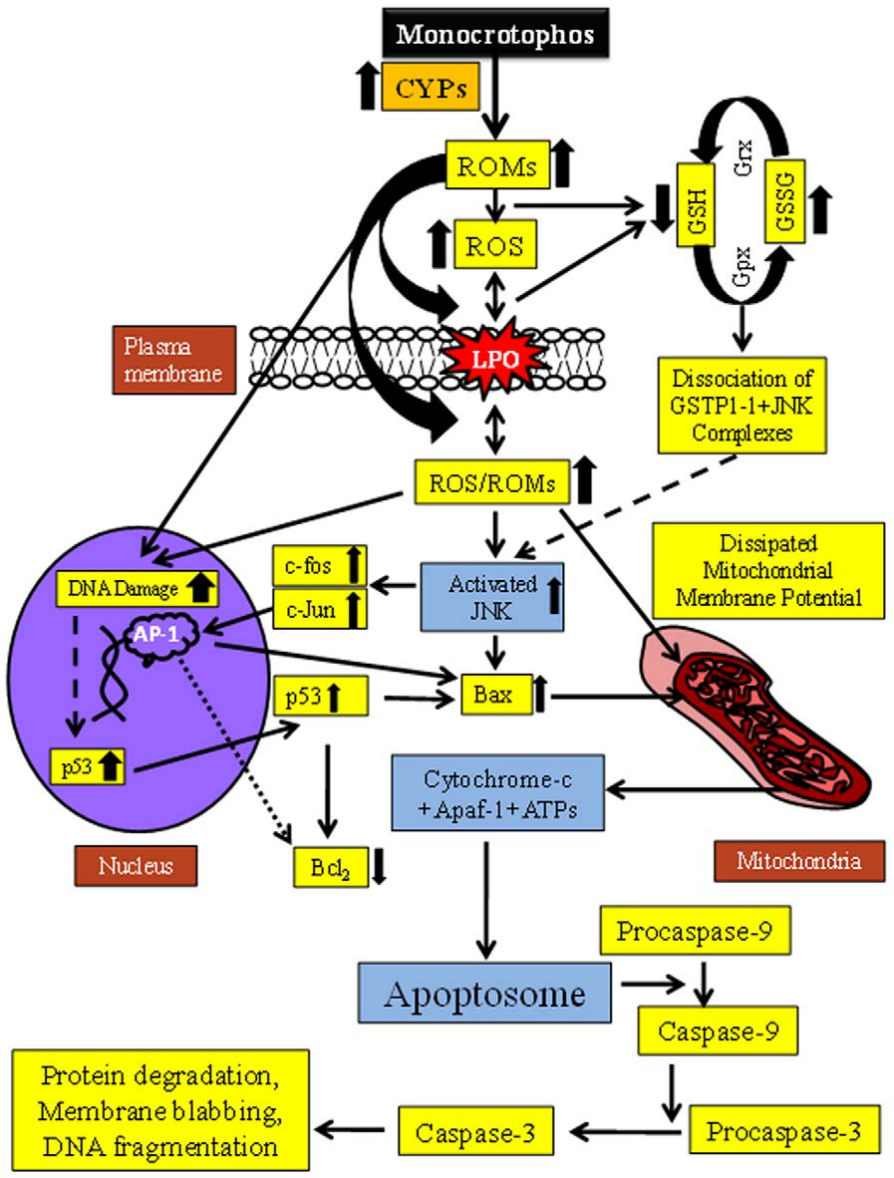
Schematic flow diagram to depict the involvement of selected CYPs in the induction of oxidative stress and apoptosis in PC12 cells expose to MCP.

In the present investigation, synchronization was also observed between the increased expressions of CYPs (1A1, 1A2, 2B1, 2B2, and 2E1) and altered expressions of caspase 3 and caspase 9, genes involved in apoptosis signalling cascade in PC12 cells. The caspase cascade activation has been reported by two different routes, i.e., binding of procaspase-9 with Apaf-1 to form the apoptosome complex following the release of cytochrome-*c* from damaged mitochondria [29], while in other route OMI, and SMACs released from intra-mitochondrial space is binds with caspase inhibitors, and thus activates the caspases [48]. But, we are hypothesizing the involvement of CYPs in the activation of caspases as another possible route to trigger the apoptosis signalling in PC12 cells receiving MCP exposure. Since, CYPs-mediated apoptotic changes have already been reported in E47 cells [49], and Hepa1c1c7 cells [24], [50] exposed to buthionine sulfoximine and Benzo[a] pyrene respectively. Based on the findings, we propose a schematic flow diagram showing the involvement of selected CYPs in the triggering of ROM induced oxidative stress and apoptosis cascade in PC12 cells exposed to MCP. Apoptosis induction was routed through mitochondrial activity and by the involvement of caspase 3/9 (Figure 8).

In summary, we believe that this is the first report showing altered expressions of selected CYPs in MCP induced apoptosis and oxidative damage in PC12 cells. These apoptotic changes were mitochondria-mediated and regulated through caspase cascade. Our data confirm the involvement of specific CYPs in MCP induced apoptosis in PC12 cells and also identifies possible cellular and molecular mechanisms of organophosphate pesticide-induced apoptosis in neuronal cells.

## Materials and Methods

### Cell culture

PC12 cells were procured from National Centre for Cell Sciences, Pune, India, and have been maintained at In Vitro Toxicology Laboratory, Indian Institute of Toxicology Research, Lucknow, India, as per the standard protocols described earlier [7]. Briefly, cells were cultured in Nutrient Mixture (F-12 Hams), supplemented with 2.5% fetal bovine serum (FBS), 15% horse serum (HS), 0.2% sodium bicarbonate (NaHCO_3_), 100 units/mL penicillin G sodium, 100 µg/mL streptomycin sulphate, and 0.25 µg/mL amphotericin B. Cultures were maintained at 37°C in 5% CO_2_-95% atmosphere under high humid conditions. Culture medium was changed twice weekly and cultures were passaged at a ratio of 1∶6 once a week. Prior to experiments, cells were screened for integrity and neuronal markers as described earlier [27]. Cells were also checked for their viability using trypan blue dye exclusion assay, and batches showing more than viability 95% were only used in the experimentation.

### Reagents and consumables

All the specified chemicals, primers, probes and reagents, viz., MCP (Dimethyl (E)-1-methyl-2-methyl carbanoyl vinyl phosphate (IUPAC) C7H14NO5-P.; Catalog no. PS-609; purity-99.5%), and diagnostic kits were purchased from Sigma, USA, unless otherwise stated. Culture medium nutrient mixture F-12 Hams, antibiotics/antimycotics, fetal bovine and horse sera were purchased from Gibco BRL, USA.

### Selection of noncytotoxicity doses

Non-cytotoxic doses of monocrotophos (MCP) were identified using standard endpoints of cytotoxicity, i.e., MTT [3-(4, 5-dimethylthiazol-2-yl)-2, 5-diphenyltetrazoliumbromide], NRU (neutral red uptake), LDH (lactate dehydrogenase) released, and trypan blue assays. The selection of MCP concentrations for the present investigations was based on our previous studies with same cell line under identical conditions [7].

### Estimation of Glutathione (GSH) levels

Glutathione (GSH) levels were assessed following the exposure of MCP (10^−4^–10^−7^ M) to PC12 cells for 6, 12, and 24 h using commercially available kit (Glutathione Detection Kit, Catalog no. APT250, Chemicon, USA). In brief, following respective MCP exposures, cells were collected by centrifugation at 700×g for 2 min at 4°C and lysed in lysis buffer. The samples were centrifuged again at 12,000×g for 10 min at 4°C and supernatant was collected. To estimate the GSH levels, the lysed samples (90 µl/well) were transferred to 96 well black bottom plates and mixed with freshly prepared assay cocktail (10 µl) and read at excitation wavelength 380 nm and emission wavelength 460 nm using Multiwell Microplate Reader (Synergy HT, Bio-Tek, USA) after the incubation of 1–2 h. Standard curve was plotted using the glutathione standard supplied in the kit and used to calculate the experimental values. Cells exposed to H_2_O_2_ (100 µM) for 2 h under identical conditions were served as positive control.

### Estimation of Reactive Oxygen Species (ROS) generation

Estimation of MCP-induced ROS generation was carried out following the standard protocol of Srivastava et al. [51]. In brief, cells (1×10^4^ per well) were seeded in poly L-lysine pre-coated 96 well black bottom culture plates and allowed to adhere for 24 h in 5% CO_2_–95% atmosphere at 37°C. Cells were exposed to MCP (10^−4^ to 10^−8^ M) for 6, 12 and 24 h. Following the exposure, cells were re-incubated with 2′, 7′ dichloro-dihydrofluorescein-diacetate (DCFH-DA) (20 µM) for 30 min at 37°C. The reaction mixture was then replaced by 200 µl of PBS per well. The plates were kept on rocker shaker platform for 10 min at room temperature in dark and fluorescence intensity was measured using multiwall micro plate reader (Synergy HT, Bio-Tek, USA) on excitation wavelength at 485 nm and emission wavelength at 528 nm. The data are expressed in percent of the unexposed control.

Intracellular ROS generation was also confirmed by image analysis. In brief, cells (5×10^4^ per well) were seeded in Poly L- Lysine pre-coated tissue culture slide flasks and allowed to adhere. Adhered cells were then exposed to MCP (10^−5^ M to 10^−7^ M) for 6 h. Following MCP exposure, cells were washed twice with PBS, and re-incubated for 30 min in dark in incomplete culture medium containing DCFH-DA (20 µM). Slides were washed twice again with PBS and mounted for microscopic analysis. Images were taken by using Nikon Eclipse 80i equipped with Nikon DS-Ri1 12.7 megapixel camera. Cells exposed to H_2_O_2_ (100 µM) for 2 h under identical conditions were served as positive control.

### Detection of apoptosis

Mitochondrial membrane potential, an early marker of apoptosis induction was assessed using Flowcytometer based Mitolight™ Apoptosis Detection Kit (APT142, Chemicon, USA). Cells were exposed to MCP (10^−4^ M-10^−6^ M) for 6 h, then pelleted and re-suspended in 1 ml of pre-diluted Mitolight™ solution for 15 min at 37°C. Following incubation, uptake of Mitolight™ dye by living mitochondria was analyzed by Flowcytometer (BD FACSCanto™) equipped with the FACS Diva Version 6.0.0.software. Validation of data was also done using fluorescence microscopy (Nikon Eclipse 80i) attached with Nikon digital CCD cool camera -Model DS-Ri1 of 12.7 Megapixel).

### Nuclear condensation by Bis-bezimide Staining

MCP-induced apoptotic alterations were also observed by counting the events of nuclear condensation. Nuclear condensation was observed under fluorescence microscope using (2′-[4-Ethoxyphenyl]-5-[4-methyl-1-piperazinyl]-2, 5′-bi-1H-benzimidazole] (Hochest no. 33342, Sigma, USA) dye as described by Srivastava et al. [51]. Data was presented by comparing the values with un-exposed control cells.

### Real Time - PCR (TaqMan Chemistry)

Expression (mRNA) of xenobiotic metabolizing cytochrome P450s (CYP1A1, 1A2, 2B1, 2B2, & 2E1) was studied in PC12 cell exposed to MCP (10^−6^ M &10^−5^ M) for 2, 6, 12, and 24 h. Total RNA was isolated using GeneElute mammalian total RNA Miniprep Kit (Catalog no. RTN-70, Sigma, USA). The quality of RNA was checked by Nanodrop ND-1000 Spectrophotometer V3.3 (Nanodrop Technologies Inc., Wilmington, DE, USA) as well as by running RNA onto 2% denaturing agarose gel. Total RNA (1 µg) was reverse-transcribed into cDNA by SuperScript III first strand cDNA synthesis Kit (Catalog no. 18080-051, Invitrogen Life Science, USA) using random hexamer primers. Quantitative Real Time PCR (RT-PCR^q^) was performed in 96 well plate format using TaqMan primers and probes in ABI PRISM® 7900HT Sequence Detection System (Applied Biosystems, USA). The TaqMan 20 µl reaction mixture contained 1 µl of 4 µM probe (final concentration, 0.2 µM), 1 µl of 10 µM forward primer and 1 µl of 10 µM reverse primer (0.5 µM final concentration for each primer), 10 µl of TaqMan Universal master mix, 6 µl of nuclease-free water, and 1 µl of cDNA (50 ng of total RNA). After sealing the plate with an optical adhesive cover, the thermo-cycling conditions were initiated at 50°C for 2 min with an enzyme activation step of 95°C for 10 min followed by 40 PCR cycles of denaturation at 95°C for 15 seconds and anneal/extension at 60°C for 1 min. Sequence of primers and probes used were: CYP1A1 (M26129a) forward 5′-ccaaacgagttccggcct-3′, reverse 5′- tgcccaaaccaaagagaatga-3, probe 5′-ttctcactcaggtgtttgtccagagtgcc-3′; CYP1A2 (K02422a) forward 5′-cgcccagagcggtttctta-3′, reverse 5′-tcccaagccgaagagcatc-3′, probe 5′-caatgacaacacggccatcgacaag-3′; CYP2B1 (J00719a) forward 5′- aacccttgatgaccgcagtaaa-3′, reverse 5′- tgtggtactccaatagggacaagatc-3′, probe 5′-ccatacactgatgcagttatccatgagattcaga-3′; CYP2B2 (J00720–728a) forward 5′-ccatcccttgatgatcgtacca-3′, reverse 5′-aattggggcaagatctgcaaa-3′ probe 5′-ccatacactgatgcagtcatccacgagattc-3′, CYP2E1 (J02627a) forward 5′-aaagcgtgtgtgtgttggagaa-3′, reverse 5′-agagacttcaggttaaaatgctgca-3′, probe 5′-atagcagacaggagcagaaacaattccatgc-3′ and Actin-β (V01217) forward 5′- ggaaatcgtgcgtgacattaaag-3′, reverse 5′- cggcagtggccatctctt-3′, probe 5′-agctgtgctatgttgccctagacttcgagc-3′. Actin-β was used as internal control to normalize the data. MCP-induced alterations in mRNA for specific CYPs are expressed in relative quantity keeping values of unexposed control groups as basal, i.e., one. Real time reactions were carried in triplicate well for each sample.

### Real Time - PCR (SYBR Green Chemistry) analysis

Alterations in mRNA expression of oxidative stress and apoptosis markers were studied in PC12 cells exposed to MCP (10^−5^ M) for 6 h. The methodology was same as used for mRNA expression of CYPs except the use of SYBR Green instead of TaqMan probes. Specificity of primer sets and genomic DNA contamination were assessed for all the samples by analyzing by melting curve analysis and running no template control (NTCs). The primer sequences used in the study were similar as reported by us earlier [7].

### Western blot analysis

Western blot analysis was carried out for xenobiotic metabolizing CYP P450s (CYP1A1, 1A2, 2B1, 2B2, & 2E1), oxidative stress and apoptosis markers (GSTP1-1, p53, Bax, Bcl_2_, activated Caspase-9, & activated Caspase-3) in PC12 cells exposed to MCP (10^−5^ M) for 6, 12, and 24 h. Following MCP exposure, cells were pelleted and lysed using CelLytic™ M Cell Lysis Reagent (Catalog no. C2978, Sigma, USA) in the presence of protein inhibitor cocktail (Catalog no. P8340-5ML, Sigma, USA). Protein estimation was done by BCA Protein Assay Kit (Catalog no. G1002, Lamda Biotech, Inc., St. Louise, MO, USA). The equal amount (50 µg/well) of denatured proteins was loaded in 10% tricine-SDS gel and blotted on polyvinylidene fluoride (PVDF) membranes (Santa Cruz, USA) using wet transfer system. After blocking (2 h at 37°C), membranes were incubated overnight at 4°C with anti-protein primary antibodies specific for 1A1, 1A2, 2B1/2B2 & 2E1 (1∶500, Chemicon, USA), GSTP1-1 (1∶1000, Calbiochem, USA), p53, Bcl_2_, Bax, Activated Caspase-9, Activated Caspase- 3 (1∶1000, CST, USA) and Actin-β (1∶2000, Santa Cruz, USA) in blocking buffer (pH 7.5). The membranes were then re-incubated for 2 h at room temperature with secondary anti-primary immunoglobulin G (IgG)-conjugated with horseradish peroxidase (Calbiochem, USA). The blots were developed using luminol (Catalog no. 34080, Thermo Scientific, USA) and densitometry was done for protein specific bands in Gel Documentation System (Alpha Innotech, USA) having AlphaEase™ FC StandAlone V. 4.0.0 software. Actin-β was used as internal control to normalize the data. MCP induced alterations are expressed in relative term fold change in expression by comparing the data with respective unexposed controls. Auto-recovery pattern of altered protein levels was also studied in a parallel group exposed to MCP (10^−5^ M) for 6 h followed by 18 h incubation in fresh culture medium without MCP.

### Immunocytochemical analysis

Immunocytochemical localization of early response marker proteins such as c-fos and c-jun was carried out by using anti-primary antibodies following the protocol of Siddiqui et al. [52]. Briefly, cells (1×10^4^ cells/well) were allowed to adhere on the surface of Poly L-lysin pre-coated eight well chamber slides. Cells were exposed to MCP (10^−5^ M and 10^−6^ M) for 6 h. Following exposure, cells were fixed by using 4% paraformaldehyde for 10 min and blocked with PBS containing 0.02% Triton-X100 and 0.1% BSA for 2 h to block the non-specific binding sites. Cells were then incubated with primary antibodies, viz., C-fos (1∶200, Santa Cruz, USA) and C-jun (1∶200, Santacruz, USA) for 2 h at room temperature followed by washing with PBS. Cells were re-incubated with HRP conjugated goat anti-rabbit secondary antibody (Calbiochem, USA) for 2 h at room temperature. Finally, cells were washed with PBS to remove unbound antibody and incubated with DAB (diaminobezidiene tetrahydrochloride, Sigma, USA) for 5–15 min to develop the brown color. Cells were visualized under upright microscope (Nikon Eclipse 80i equipped with Nikon DS-Ri1 12.7 megapixel camera, Japan) and quantification was done by measuring the change in percent area of protein expression with the help of Leica Qwin 500 Image Analysis Software (Leica, Germany).

### Statistical analysis

Results were expressed as mean ± standard error of mean (SEM) for the values obtained from at least three independent experiments. Statistical analysis was performed using one-way analysis of variance (ANOVA) and post hoc Dunnett (two sided) test to compare the findings in different groups. The values p<0.05 were considered significant.
